# Perceived stress, coping strategies, HRV-based stress index, and attentional performance in tunnel control room operators: a pilot descriptive study

**DOI:** 10.3389/fpsyg.2026.1876583

**Published:** 2026-10-20

**Authors:** Eliška Šimůnková, Eva Kozáková, Zdeněk Hon

**Affiliations:** 1 Department of Health Care Disciplines and Population Protection, Faculty of Biomedical Engineering, Czech Technical University in Prague, Czechia; 2 Department of Security Management, Ambis University, Prague, Czechia; 3 Department of Applied Social Sciences and Humanities, University of Defence, Brno, Czechia

**Keywords:** coping strategies, device-generated HRV-based stress index, human factors, perceived stress, safety-critical occupations, selective attention, tunnel control room operators

## Abstract

Safety-critical occupations place sustained demands on stress regulation, attentional functioning, and work performance under conditions of operational responsibility and time pressure. The aim of this pilot descriptive study was to examine perceived stress, coping strategies, attentional performance, and a device-generated HRV-based stress index in tunnel control room operators during a 12-h day shift. The study sample consisted of 30 male operators working in a road tunnel control centre. Perceived stress was assessed using the Perceived Stress Scale (PSS-10), coping strategies using the Stressverarbeitungsfragebogen (SVF-78), and selective attention performance using the d2 Test of Attention. The device-generated HRV-based stress index was recorded during sequential mid-shift and end-shift measurement blocks. Concentration Performance scores were measured at the beginning and end of the shift. Paired-samples t-tests were used for repeated measurements. Most participants were classified in the moderate perceived stress category. The device-generated HRV-based stress index was significantly higher during the end-shift measurement block than during the mid-shift measurement block. However, because no start-of-shift HRV baseline was obtained and time-of-day and meal-related factors were not controlled, this difference cannot be attributed specifically to cumulative shift-related strain. No statistically significant change in Concentration Performance scores was identified. The highest coping-strategy means were observed for Situation Control, Reaction Control, and Positive Self-Instructions. The findings provide a preliminary multidimensional account of occupational functioning in a safety-critical environment. The device-specific HRV-based index was treated as an exploratory output and should not be interpreted as a diagnostic measure of physiological stress. Given the pilot design, small single-site sample, and methodological limitations, the findings should be interpreted cautiously and regarded as a basis for further research.

## 1 Introduction

Safety-critical work environments represent a specific context in which stress regulation, attentional functioning, and performance under occupational demands are highly relevant to stable human functioning. In such systems, the quality of decision-making is determined not only by professional expertise but also by the ability to regulate emotional activation, maintain selective attention, and manage stress effectively under conditions of time pressure and responsibility (Derdowski and Mathisen, 2023; van de Merwe et al., 2024). In this context, the human factor forms an integral part of the safety chain.

Chronic occupational stress is understood in the current literature as the result of an imbalance between job demands and available resources (Bakker and Demerouti, 2017). Long-term exposure to psychosocial risks is associated with reduced job satisfaction, increased error rates, and a higher risk of burnout (Aronsson et al., 2017; Salvagioni et al., 2017). Perceived stress, coping strategies, and attentional performance therefore represent relevant and complementary domains for examining occupational functioning under sustained demands.

A specific occupational group in this context is represented by tunnel control room operators. Their role involves continuous supervision of technological and safety systems, interpretation of operational deviations, and coordination of the response to emergency situations (Švarcová et al., 2015). Permanent International Association for Road Congresses (PIARC) recommendations emphasise the need for systematic selection and training of road tunnel operational staff, taking into account the demands of the environment and responsibility for traffic safety (PIARC, 2007). The importance of the human factor in tunnel operations is further confirmed by research integrating human performance into road tunnel risk analysis (Kirytopoulos et al., 2014).

Road tunnel safety is the outcome of a functioning socio-technical system that integrates technological infrastructure, organisational structures, and human decision-making. Directive 2004/54/EC establishes minimum safety requirements for tunnels in the trans-European road network and highlights the importance of operational and organisational components of safety (European Parliament and Council, 2004). Within a broader framework, some tunnels may form part of critical infrastructure under Directive (EU) 2022/2557 on the resilience of critical entities (European Parliament and Council, 2022). In such settings, stress regulation, attentional functioning, and operator performance are relevant not only to individual occupational well-being but also to the safe and continuous operation of transport systems.

Despite growing interest in occupational stress and the human factor in safety-critical professions, empirical research focusing specifically on tunnel control room operators remains limited. In particular, studies combining perceived stress, a device-generated HRV-based stress index, selective attention performance, and coping strategies during a real work shift are scarce. Such an approach may contribute to a more detailed understanding of stress regulation and occupational functioning under conditions of continuous supervision.

The present pilot descriptive study therefore examined perceived stress (PSS-10), coping strategies (SVF-78), a device-generated HRV-based stress index, and selective attention performance measured using the d2 Test of Attention in tunnel control room operators during a 12 h day shift. The aim was to provide a preliminary descriptive account of selected indicators related to stress regulation and attentional functioning in a safety-critical environment.

On this basis, the following research questions were formulated:

1 Which coping strategies do tunnel control room operators use to manage occupational stress?
2 Does selective attention performance differ between the beginning and the end of the shift?
3 Does the device-generated HRV-based stress index differ between the mid-shift and end-shift assessment blocks?
4 What is the level of perceived stress (PSS-10) in the occupational group under study?

## 2 Materials and methods

This section describes the methodological framework of a pilot descriptive field study focused on perceived stress, coping strategies, selective attention performance, and a device-generated HRV-based stress index in tunnel control room operators during a real work shift. The research procedure was designed to assess the selected variables in their natural occupational context while maintaining comparable measurement conditions. The study combined psychometric, performance-based, and device-generated measures to provide a multidimensional description of occupational functioning in a safety-critical environment.

### 2.1 Study design

The study was a pilot descriptive field study conducted in the natural working environment of tunnel control room operators. The research design was based on the assumption that stress in safety-critical systems is multidimensional and that its adequate assessment (Tušer et al., 2021) requires a combination of subjective, performance-based, and device-generated indicators.

Four domains were examined:

1 Perceived stress (PSS-10);
2 Coping strategies (SVF-78);
3 Selective attention performance (d2 Test of Attention);
4 Device-generated HRV-based stress index.

This multi-source approach is consistent with recommendations in the literature on stress and performance in occupational settings, which emphasises the importance of combining psychometric and physiological indicators (Cohen et al., 1983; Lee, 2012; Quintana and Heathers, 2021).

The pilot phase had two aims:

1 To verify the applicability of the selected instruments in the context of road tunnel operations;
2 To obtain a preliminary descriptive account of perceived stress, the device-generated HRV-based stress index, attentional performance, and coping strategies during a 12 h shift.

### 2.2 Participants

The study sample consisted of 30 tunnel control room operators working in road tunnel operations. The study was conducted at one of three road tunnel control centres considered for participation. Data collection took place between October 2025 and January 2026.

The sampling procedure was non-probability based and relied on the availability of respondents in their natural working environment. This approach was consistent with the pilot nature of the study and at the same time reflected the limited size and specific accessibility of the occupational group under investigation.

The inclusion criteria were as follows:

1 Employment as a tunnel control room operator in tunnel operations;
2 Active shift work;
3 Consent to the full administration of all instruments.

The aim was not to generalise the findings to the entire occupational population, but to provide a methodological verification of the research procedure. The study sample consisted exclusively of men aged 34 to 65 years (M = 51.00, SD = 9.26). All participants had at least three years of work experience and previous experience of shift work. Individual years of occupational experience were not systematically recorded. These characteristics reflected the staffing profile of the selected control centre. The recruitment strategy sought to include all tunnel control room operators employed at the selected control centre. No additional exclusion criteria were specified beyond the stated inclusion criteria because recruitment aimed to include the full available operator population at the workplace. Sleep quality, caffeine intake, and relevant health-related factors were not assessed. All 30 participants completed all study procedures.

### 2.3 Measures

#### 2.3.1 Perceived stress

Perceived stress was measured using the Perceived Stress Scale (PSS-10) (Cohen et al., 1983). The instrument assesses the extent to which an individual has perceived their life as unpredictable, uncontrollable, and overwhelming during the previous period, specifically over the past month. The PSS-10 is one of the most widely used instruments for measuring perceived stress, and its psychometric properties have been repeatedly confirmed (Lee, 2012).

The total score ranges from 0 to 40 points. Before calculation of the total score, all positively worded PSS-10 items were reverse-scored. The Perceived Helplessness and Lack of Self-Efficacy subscale scores were subsequently calculated from their respective item sets. Higher total scores indicate a higher level of perceived stress.

For descriptive purposes, total PSS-10 scores were grouped into low (0–13), moderate (14–26), and high (27–40) perceived stress categories, following published interpretive ranges. These categories were used descriptively and were not treated as formal diagnostic cut-offs (Campbell et al., 2024).

#### 2.3.2 Coping strategies

Stress coping strategies were assessed using the Stressverarbeitungsfragebogen (SVF-78) questionnaire (Janke and Erdmann, 2003). The instrument identifies relatively stable ways of coping with stressful situations and distinguishes several specific coping strategies. Its administration made it possible to identify dominant strategies and their distribution within the study sample. All 13 SVF-78 subscales were scored and included in the analysis.

#### 2.3.3 Selective attention performance

Selective attention performance was measured using the d2 Test of Attention (Brickenkamp and Zillmer, 1998). The test assesses the speed and accuracy of visual discrimination, concentration, and performance stability in a time-limited task.

For the analysis, the Concentration Performance (CP) index was used, defined as the number of correctly marked items minus the number of errors. The CP index was selected because it combines speed and accuracy in a single indicator and is regarded as a relevant measure of concentrated selective attention under time-limited task conditions. In the literature, this index is regarded as a key indicator of selective attention and performance stability.

#### 2.3.4 Device-generated HRV-based stress index

The device-generated HRV-based stress index was recorded using the Max Pulse system (Medicore Co., Ltd., Seoul, Republic of Korea; device manufactured in 2013), operated with MaxPulse Software. The exact software and firmware versions were not available from the device records. The device generated a numerical composite output from pulse-wave recordings obtained using a finger-clip sensor. Heart Rate Variability (HRV) is a non-invasive indicator of autonomic nervous system activity and reflects variation in autonomic regulation (Shaffer and Ginsberg, 2017; Quintana and Heathers, 2021).

The device output was presented on a 100-point scale, with higher values indicating a higher device-generated stress index. The numerical HRV-based stress index was used in the analysis; categorical labels generated by the device were not analysed. The index was treated as an exploratory device-specific composite output. The detailed calculation algorithm and independent validation of the specific Max Pulse index were not available to the authors. Consequently, the index was not interpreted as a diagnostic measure of stress. Its analysis was limited to within-participant comparison between the two measurement points. Interpretation followed recommendations emphasising standardised measurement conditions and caution when drawing conclusions from HRV-derived indicators (Quintana and Heathers, 2021; Teferra et al., 2023).

### 2.4 Procedure

Data collection took place during regular 12-h day shifts from 06:00 to 18:00 in the participants’ natural working environment. The administration procedure was as follows:

1 the PSS-10 and SVF-78 were completed during a quiet period of the shift;
2 the d2 Test of Attention was administered twice, at the beginning and at the end of the shift;
3 the device-generated HRV-based stress index was recorded using a single Max Pulse device during two measurement blocks.

The mid-shift assessment began at approximately 11:00, five hours after shift commencement, and the end-shift assessment began at approximately 16:00, two hours before the scheduled end of the shift. Participants were assessed sequentially, and each individual recording lasted approximately 5 min. Consequently, obtaining one set of recordings from all 30 participants required approximately 150 min of total measurement time at each assessment occasion. A start-of-shift HRV assessment was not included in the original study protocol. The beginning of the shift was allocated to the pre-shift administration of the d2 Test of Attention. Because one complete sequential HRV measurement block required approximately 150 min, a third HRV block at shift commencement would have overlapped with the d2 assessment and the participants’ operational duties. The pilot protocol therefore included two HRV measurement blocks only.

For each HRV assessment, the participant was seated at a table in a quiet environment. A finger-clip sensor connected to the Max Pulse device was attached to the left index finger. Participants were instructed to remain still and refrain from speaking during the recording. Signal quality was monitored throughout data acquisition. No recordings required repetition because of insufficient signal quality, and no ectopic beats were identified in the analysed recordings.

Before each HRV assessment, participants had been performing their usual work duties. No separate standardised pre-measurement rest period was applied. Meal timing and food intake were not standardised or systematically recorded. Attentional performance was assessed between the beginning and the end of the full 12-h shift. In contrast, the device-generated HRV-based stress index was compared between two measurement blocks beginning at approximately 11:00 and 16:00. The HRV comparison therefore did not represent a change across the entire shift.

Each participant signed an informed consent form before the start of data collection. Participation was voluntary, and the data were processed anonymously.

### 2.5 Data analysis

Data analysis was performed using Microsoft Excel 2021 and STATISTICA 14. Given the pilot nature of the study, descriptive statistics constituted the primary analytical approach. Paired-samples t-tests were used to examine repeated measurements of attentional performance and the device-generated HRV-based stress index. All statistical tests were two-sided. The normality of the paired difference scores was assessed using the Shapiro–Wilk test, and potential outliers were examined using the 1.5 × interquartile range criterion.

For quantitative variables, including the total PSS-10 score, Perceived Helplessness, Lack of Self-Efficacy, Concentration Performance in the d2 Test of Attention, and the device-generated HRV-based stress index, the arithmetic mean, standard deviation, median, minimum, and maximum were calculated. The range was expressed as a min–max interval.

For the categorical variable Level of stress, absolute frequencies and percentage distributions of the individual categories were calculated.

Data from the SVF-78 questionnaire were first scored according to the original procedure of the instrument (Janke and Erdmann, 2003). Arithmetic means and standard deviations of the raw scores were subsequently calculated for all 13 subscales in the full study sample. For exploratory descriptive comparison, the sample means were compared with the corresponding male normative raw-score means reported in Table 5.5/1 of the SVF-78 manual. These normative comparisons were used to provide contextual interpretation of the observed coping profile. As no occupation-specific norms are available for tunnel control room operators, the comparisons were treated solely as descriptive references and were not used as a basis for inferential testing or population-level conclusions.

The device-generated HRV-based stress index was summarised separately for the mid-shift and end-shift assessments. Pre-test and post-test Concentration Performance (CP) scores and mid-shift and end-shift HRV-based stress index values were compared using paired-samples t-tests. Cohen’s *d*z and 95% confidence intervals were reported. Statistical significance was set at

*p* < 0.05. Analysis of the HRV-based stress index was limited to within-participant comparison between the two measurement points. All 30 participants had complete data for all study measures. No data imputation or other missing-data procedure was therefore required.

The study was reviewed by the Ethics Committee of Ambis University, a.s., which determined that formal ethical approval was not required (Statement No. EC 05/2025). The research was conducted in accordance with the principles of the Declaration of Helsinki. All participants provided written informed consent prior to participation. Participation was voluntary, and data were anonymised and processed exclusively for research purposes.

## 3 Results

This section presents the results of the pilot study, organised according to the measured variables. The results for perceived stress (PSS-10) are presented first, followed by the device-generated HRV-based stress index, selective attention performance, and coping strategies. Descriptive and inferential results are presented in Figures 1–3 and Tables 1–4.

**Figure 1:**
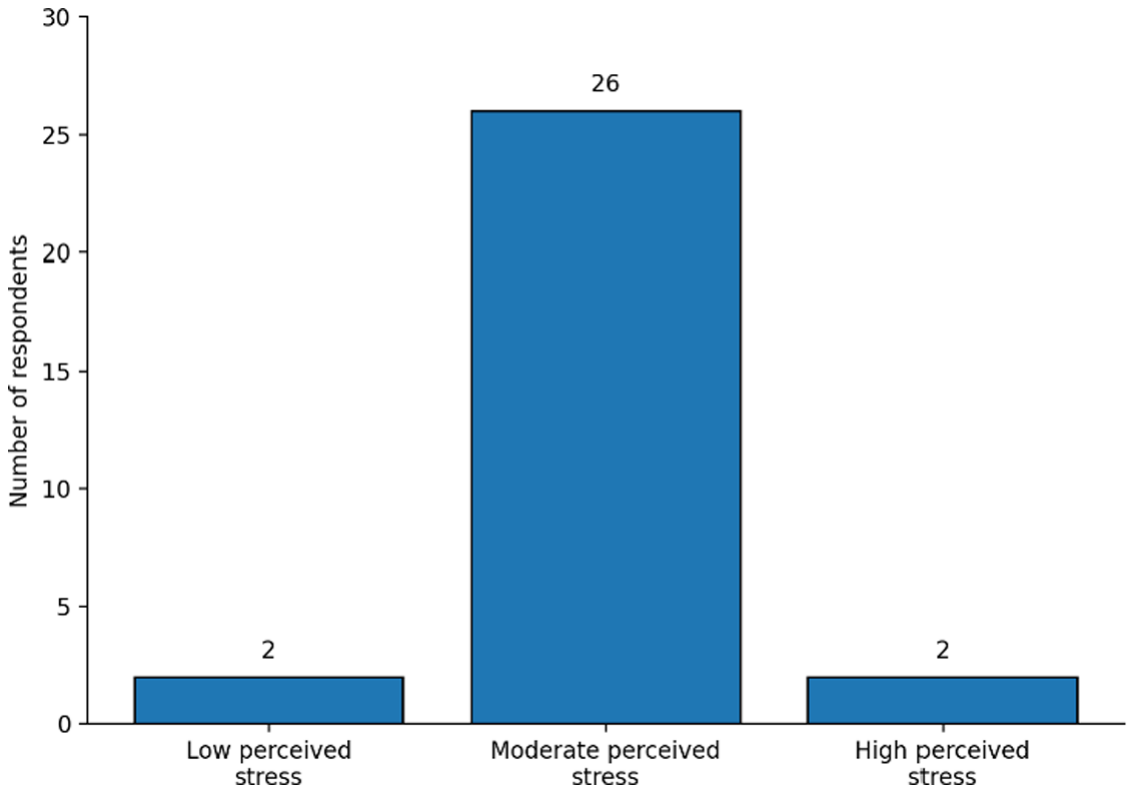
Distribution of perceived stress categories. Source: Compiled by the authors.

**Figure 2:**
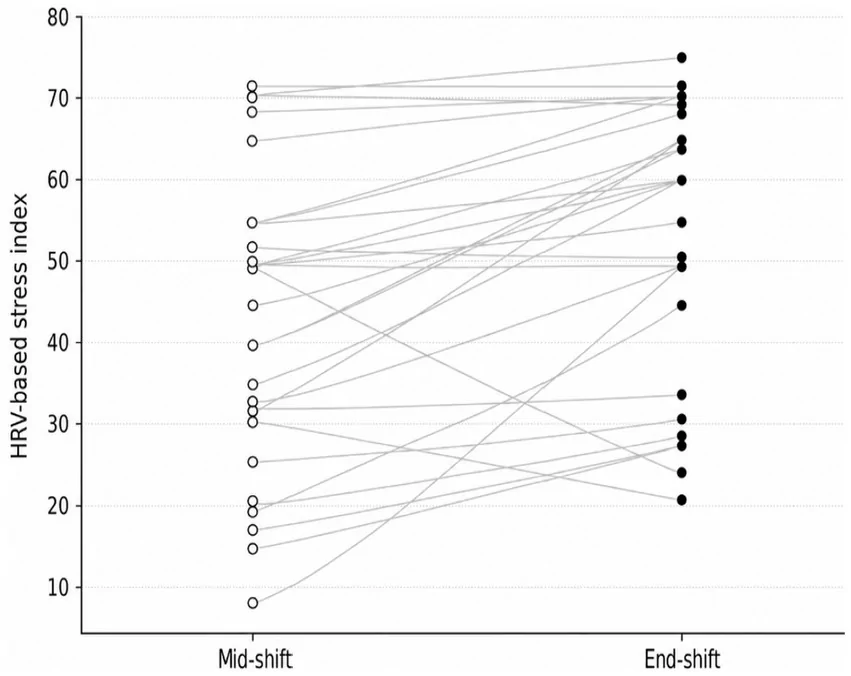
Paired device-generated HRV-based stress index values for the mid-shift and end-shift measurement blocks. Source: Compiled by the authors.

**Figure 3:**
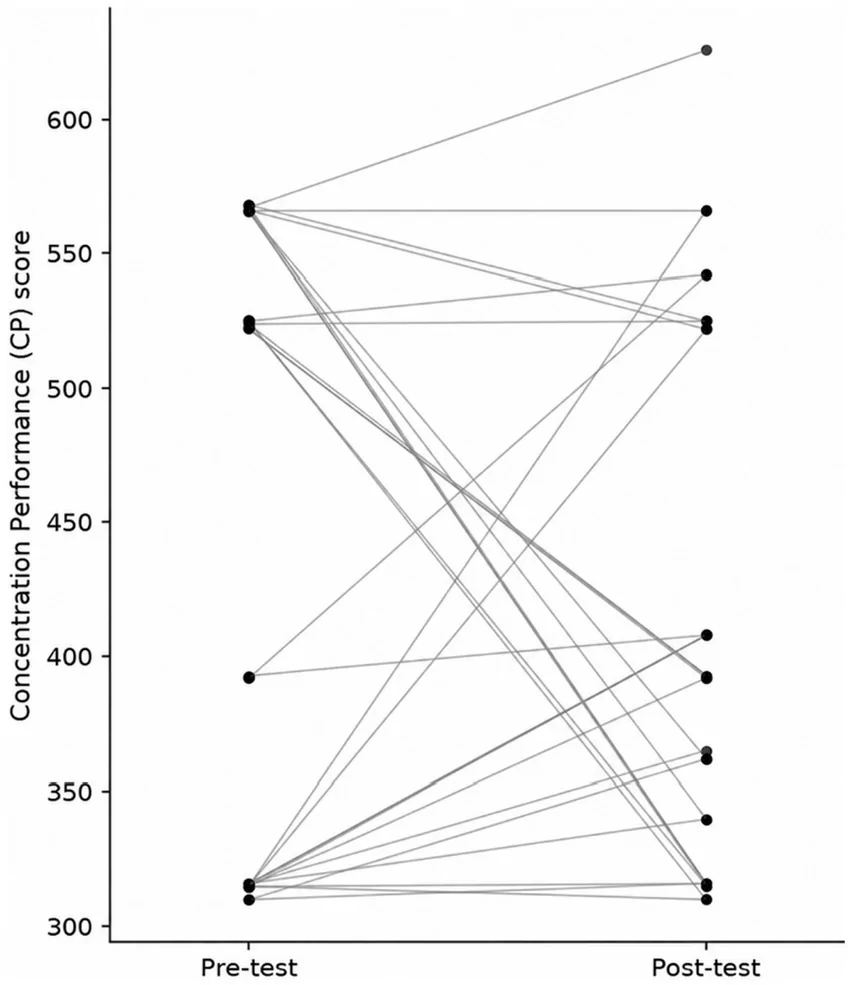
Paired pre-test and post-test concentration performance (CP) scores (N = 30). Source: Compiled by the authors.

**Table 1:** Descriptive statistics for PSS-10 total score and subscales (*N* = 30).

Variable	Mean	SD	Median	Min	Max
Total stress (PSS-10)	20.80	4.35	21	8	27
Perceived helplessness (0–24)	10.00	5.83	8	0	22
Lack of self-efficacy (0–16)	10.80	3.26	12	5	14

Source: Compiled by the authors.

**Table 2:** Descriptive statistics for the device-generated HRV-based stress index (*N* = 30).

Variable	Mean	SD	Median	Min	Max
Mid-shift measurement block	45.50	18.83	50	8	71
End-shift measurement block	54.87	16.75	60	21	74

Source: Compiled by the authors.

**Table 3:** Descriptive statistics for concentration performance (CP) scores in the d2 test of attention (*N* = 30).

Measurement	*N*	Mean	SD	Median	Min	Max
Pre-test	30	444.17	115.44	522.00	310	568
Post-test	30	414.03	96.90	392.50	310	626

Source: Compiled by the authors.

**Table 4:** Descriptive statistics and exploratory differences from male normative raw-score means for the SVF-78 subscales (*N* = 30).

Coping strategy	Mean	SD	Male normative mean (raw score)	Difference
Undervaluation	13.30	3.46	10.67	2.63
Rejection of Guilt	11.70	3.58	11.41	0.29
Diversion	12.70	3.26	11.69	1.01
Substitute satisfaction	9.23	4.30	8.42	0.81
Situation control	19.23	2.67	16.84	2.39
Reaction control	16.97	3.80	15.27	1.70
Positive self-instructions	16.90	3.26	16.71	0.19
Need for social support	12.77	5.80	11.57	1.20
Avoidance	16.60	3.23	11.44	5.16
Escape tendency	9.63	4.94	7.35	2.28
Perseveration	13.23	6.42	13.61	−0.38
Resignation	7.30	5.46	7.17	0.13
Self-blame	10.00	5.28	9.81	0.19

Difference = sample mean − male normative raw-score mean.

Source: Authors’ calculations based on the study data; male normative raw-score means from Janke and Erdmann (2003, Table 5.5/1).

### 3.1 Subjective perception of stress

Perceived stress was measured using the Perceived Stress Scale (PSS-10) (Cohen et al., 1983). The total score ranges from 0 to 40 points, with higher values indicating a higher level of perceived stress.

In the analysed sample (*N* = 30), the mean total PSS-10 score was 20.80 (SD = 4.35; median = 21; range = 8–27).

Descriptive statistics for the subscales showed the following values:

- Perceived Helplessness (0–24):

Mean = 10.00 (SD = 5.83), median = 8, range = 0–22.

- Lack of Self-Efficacy (0–16):

Mean = 10.80 (SD = 3.26), median = 12, range = 5–14.

Detailed values are presented in Table 1.

The participants were further grouped into three categories according to their level of perceived stress. The distribution was as follows:

- Low perceived stress: 2 participants (6.67%);
- Moderate perceived stress: 26 participants (86.67%);
- High perceived stress: 2 participants (6.67%).

Figure 1 presents the distribution of perceived stress categories (*N* = 30). Most participants were classified in the moderate perceived stress category (86.67%).

Descriptive statistics for the total PSS-10 score and both subscales are presented in Table 1.

### 3.2 Device-generated HRV-based stress index

The device-generated HRV-based stress index was recorded during two sequential measurement blocks beginning at approximately 11:00 and 16:00 within the 12-h day shift. Descriptive statistics for both measurement blocks are presented in Table 2. Values from the mid-shift and end-shift measurement blocks were compared using a paired-samples t-test. Cohen’s *d*z and a 95% confidence interval were reported.

Figure 2 shows individual within-participant differences in the device-generated HRV-based stress index between the mid-shift and end-shift measurement blocks. The distribution of the paired difference scores did not significantly deviate from normality (Shapiro–Wilk *W* = 0.957, *p* = 0.257). Two potential outliers were identified using the 1.5 × interquartile range criterion and were retained in the analysis. The device-generated HRV-based stress index was significantly higher during the end-shift measurement block than during the mid-shift measurement block, *t*(29) = 3.89, *p* < 0.001, Cohen’s *d*z = 0.71. The mean paired difference, calculated as end-shift minus mid-shift, was 9.37 points (95% CI [4.44, 14.29]).

### 3.3 Attentional performance

Selective attention performance was assessed using the d2 Test of Attention. The concentration index (Concentration Performance; CP) was selected as the main indicator. This index represents the difference between the number of correctly marked items and the number of errors.

Measurement was carried out at two time points during the 12-h day shift: at its beginning (pre-test) and at its end (post-test). The analysis included the entire study sample (*N* = 30). Descriptive statistics for the CP scores are presented in Table 3. Pre-test and post-test scores were compared using a paired-samples t-test. Cohen’s dz and a 95% confidence interval were reported.

The mean pre-test CP score was 444.17 (SD = 115.44; median = 522.00; range = 310–568). The mean post-test CP score was 414.03 (SD = 96.90; median = 392.50; range = 310–626).

Paired differences were calculated as post-test minus pre-test; therefore, negative values indicate a lower CP score at post-test relative to pre-test.

Paired pre-test and post-test CP scores are shown in Figure 3. The distribution of the paired difference scores did not significantly deviate from normality (Shapiro–Wilk *W* = 0.939, *p* = 0.086), and no potential outliers were identified using the 1.5 × interquartile range criterion. Although the mean CP score was lower at post-test, the paired difference was not statistically significant, *t*(29) = −1.18, *p* = 0.248, Cohen’s *d*z = −0.22. The mean paired difference, calculated as post-test minus pre-test, was −30.13 points (95% CI [−82.43, 22.16]). No statistically significant within-participant change in CP scores was identified between the two measurement occasions.

### 3.4 Stress coping strategies of participants

Coping strategies were assessed using the SVF-78 questionnaire in the full study sample (*N* = 30). For each of the 13 subscales, the mean and standard deviation of the raw scores were calculated. Exploratory differences were calculated as the sample mean minus the corresponding male normative raw-score mean reported by Janke and Erdmann (2003, Table 5.5/1).

The highest sample mean was observed for Situation Control (*M* = 19.23, *SD* = 2.67), followed by Reaction Control (*M* = 16.97, *SD* = 3.80) and Positive Self-Instructions (*M* = 16.90, *SD* = 3.26). Self-Blame had a sample mean of 10.00 (*SD* = 5.28). The largest positive descriptive differences from the male normative raw-score means were observed for Avoidance (+5.16), Undervaluation (+2.63), and Situation Control (+2.39). Perseveration was the only subscale showing a negative descriptive difference (−0.38).

The Difference column in Table 4 represents the arithmetic difference between the sample mean and the corresponding male normative raw-score mean. These comparisons were descriptive and exploratory; no inferential comparisons with the male normative sample were performed.

## 4 Discussion

The study provides a preliminary account of perceived stress, the device-generated HRV-based stress index, selective attention performance, and coping strategies in tunnel control room operators working in a safety-critical environment. The measures represent complementary but non-equivalent indicators assessed through self-report, performance testing, and a device-generated HRV-based output. Their combined use provides a multidimensional description of occupational functioning during a 12-h day shift, but does not establish causal relationships between the measured domains.

### 4.1 Perceived stress and the device-generated HRV-based stress index

Most participants were classified in the moderate perceived stress category, whereas the device-generated HRV-based stress index was significantly higher during the end-shift measurement block than during the mid-shift measurement block. The two measures should not be interpreted as interchangeable. The Perceived Stress Scale (PSS-10) assesses subjective appraisal of stress over the preceding month (Cohen et al., 1983; Cohen and Williamson, 1988), whereas HRV-derived measures reflect short-term variation in cardiac intervals and may be influenced by autonomic, behavioural, and contextual factors (Shaffer and Ginsberg, 2017; Quintana and Heathers, 2021; Teferra et al., 2023).

The observed difference should be interpreted as an exploratory observation of temporal variability in a device-generated output under real working conditions. It does not demonstrate an increase in physiological stress or identify the physiological mechanism underlying the change. Because no start-of-shift baseline was obtained and the two measurement blocks began at different times of day, the difference cannot be attributed specifically to cumulative shift-related strain. Meal timing and food intake were not standardised or systematically recorded. Time-of-day variation, postprandial autonomic effects, operational workload, fatigue, and other unmeasured contextual factors may therefore have contributed to the observed values (Quintana and Heathers, 2021; Teferra et al., 2023). Previous studies indicate that HRV parameters may vary according to time of day and following food intake (Bonnemeier et al., 2003; Lu et al., 1999). The principal contribution of the present finding is therefore methodological: it indicates the need to validate the device output against transparent HRV metrics and to control relevant temporal, behavioural, and operational variables before physiological or operational conclusions can be drawn (Quintana and Heathers, 2021; Teferra et al., 2023).

### 4.2 Attentional performance during the shift

Selective attention represents an important component of human performance in safety-critical environments, where operators must continuously monitor information and respond to deviations (van de Merwe et al., 2024). In the present study, the mean Concentration Performance (CP) score was lower at post-test than at pre-test, but the paired difference was not statistically significant and the effect size was small. The findings therefore do not demonstrate a systematic within-participant change in selective attention during the shift. Interpretation should also account for the possibility that repeated administration of the d2 Test of Attention introduced a practice effect that may have interacted with fatigue, time of day, or workload-related influences. Tunnel control room operators form part of a socio-technical system in which human performance interacts with technological and organisational components (Kirytopoulos et al., 2014). European tunnel-safety requirements likewise recognise the importance of operational and organisational factors in addition to structural measures (European Parliament and Council, 2004). Attentional functioning is therefore relevant to tunnel operation, but the present pilot study did not measure operational errors, incidents, or system reliability. No system-level conclusions can consequently be drawn from the observed CP scores.

### 4.3 Coping strategies in the occupational context

Situation Control, Reaction Control, and Positive Self-Instructions showed the highest sample means. The descriptive profile is compatible with coping directed towards appraisal of the situation and regulation of individual responses. Within the transactional model of stress, problem-focused coping is particularly relevant when a person perceives that the demands of a situation can be influenced (Lazarus and Folkman, 1984).

The exploratory comparison with the male normative raw-score means showed the largest positive descriptive differences for Avoidance, Undervaluation, and Situation Control. Perseveration was the only subscale showing a small negative difference, while Self-Blame was close to the corresponding male normative mean. These differences were not tested inferentially and should not be interpreted as evidence that tunnel control room operators differ systematically from the male normative population. The comparatively higher descriptive value for Avoidance should therefore be regarded as a hypothesis-generating observation requiring confirmation in larger samples.

Coping should also be interpreted in relation to the organisational environment. Occupational stress can be influenced by job demands, available resources, shift arrangements, and opportunities for social support (Bakker and Demerouti, 2017; Załuski and Makara-Studzińska, 2022). Long-term imbalance between demands and resources may contribute to adverse occupational outcomes, including burnout (Aronsson et al., 2017; Salvagioni et al., 2017; Edú-Valsania et al., 2022). The present findings do not establish long-term occupational sustainability, but identify coping domains that may warrant further investigation in tunnel control room operations.

### 4.4 Implications for further assessment and training

Stress-management skills, coping strategies, and the maintenance of attentional performance are relevant components of professional preparation in safety-critical systems. Occupational education and training may therefore include approaches supporting stress regulation, situational control, and sustained attention under demanding working conditions (Tušer, 2020).

The findings of the present study support the value of a multidimensional assessment of workload that includes subjective, performance-based, and device-generated indicators. Such an approach may help identify areas for further assessment in safety-critical occupational settings. Given the pilot and descriptive nature of the study, the present data do not provide a sufficient empirical basis for specific training design.

### 4.5 Methodological aspects

A strength of the pilot field design was the collection of psychometric, performance-based, and device-generated data in the participants’ natural working environment. The multimethod approach reduced reliance on a single type of measurement and enabled repeated within-participant comparisons for attentional performance and the HRV-based index.

The measures nevertheless differed in their reference periods and interpretative scope. The PSS-10 referred to the preceding month, the d2 Test of Attention assessed performance at two points during one shift, and the Max Pulse output was based on approximately 5-min recordings. Direct correspondence among these indicators should therefore not be assumed. HRV-derived findings also require standardised measurement conditions and cautious interpretation because cardiac variability can be influenced by numerous physiological and contextual factors (Quintana and Heathers, 2021; Teferra et al., 2023).

### 4.6 Study limitations

The pilot study was conducted with a small non-probability sample of 30 male operators from a single control centre and included only 12-h day shifts. These characteristics limit the generalisability of the findings to female operators, other control centres, the wider occupational population, and night-shift conditions.

The HRV protocol comprised two sequential measurement blocks without a start-of-shift baseline or continuous monitoring. The beginning of the shift was allocated to the pre-shift d2 assessment, and each complete HRV block required approximately 150 min; a third block would therefore have overlapped with testing and operational duties. Participants performed their usual work duties before measurement, no standardised pre-measurement rest period was applied, and meal timing and food intake were not standardised or systematically recorded. Sleep quality, caffeine intake, recent physical activity, prior fatigue, traffic intensity, and operational incidents were also not systematically assessed. Consequently, the observed difference may have been influenced by time-of-day, postprandial, workload-related, fatigue-related, and other contextual factors and cannot be interpreted as evidence of a shift-related increase in physiological stress (Quintana and Heathers, 2021; Teferra et al., 2023; Bonnemeier et al., 2003; Lu et al., 1999).

Interpretation is further limited by the proprietary calculation algorithm and the absence of independent validation of the specific Max Pulse composite index. The observed difference should therefore be regarded as an exploratory change in a device-generated output rather than as diagnostic evidence of physiological stress (Shaffer and Ginsberg, 2017; Quintana and Heathers, 2021).

Repeated administration of the d2 Test of Attention may have introduced a practice effect that interacted with fatigue, workload, and individual variability. Future studies should include larger multisite samples, day- and night-shift measurements, start-of-shift physiological baselines, independently validated physiological measures, standardised pre-measurement conditions, and systematic assessment of relevant contextual variables.

## 5 Conclusion

This pilot descriptive study provides a multidimensional account of perceived stress, coping strategies, attentional performance, and a device-generated HRV-based stress index in tunnel control room operators. Most participants were classified in the moderate perceived stress category, while Situation Control, Reaction Control, and Positive Self-Instructions showed the highest coping-strategy means. No statistically significant within-participant change in attentional performance was identified. The proprietary device-generated index differed between the mid-shift and end-shift measurement blocks; this finding should be interpreted as an exploratory change in a device output rather than as evidence of increased physiological stress.

The findings should be interpreted cautiously because of the small single-site sample, the differing measurement intervals, and the limited independent validation of the device-specific index. Further research using larger multisite samples, transparent and independently validated physiological measures, start-of-shift baselines, and systematic assessment of temporal and contextual factors is required before operational implications can be established.

## Data Availability

The raw data supporting the conclusions of this article will be made available by the authors, without undue reservation.
